# Comparison of the extracellular vesicle proteome between glaucoma and non-glaucoma trabecular meshwork cells

**DOI:** 10.3389/fopht.2023.1257737

**Published:** 2023-10-06

**Authors:** Fiona S. McDonnell, Bre’Ida J. Riddick, Haven Roberts, Nikolai Skiba, W. Daniel Stamer

**Affiliations:** ^1^ Department of Ophthalmology, University of Utah Medical Center, Salt Lake City, UT, United States; ^2^ Department of Biomedical Engineering, University of Utah Medical Center, Salt Lake City, UT, United States; ^3^ Department of Ophthalmology, Duke University, Durham, NC, United States; ^4^ PreciseBio, Winston-Salem, NC, United States; ^5^ Department of Biological Sciences, North Carolina State University, Raleigh, NC, United States

**Keywords:** glaucoma, trabecular meshwork, extracellular matrix, extracellular vesicles, conventional outflow pathway

## Abstract

**Introduction:**

Extracellular matrix (ECM) materials accumulate in the trabecular meshwork (TM) tissue of patients with glaucoma, which is associated with a decrease in aqueous humor outflow and therefore an increase in intraocular pressure. To explore a potential mechanism for ECM regulation in the TM, we purified extracellular vesicles (EVs) from conditioned media of differentiated TM cells in culture isolated from non-glaucomatous and glaucomatous human donor eyes.

**Methods:**

EVs were purified using the double cushion ultracentrifugation gradient method. Fractions containing EV markers CD9 and TSG101 were analyzed using nanoparticle tracking analysis to determine their size and concentration. We then determined their proteomic cargo by mass spectrometry and compared protein profiles of EVs between normal and glaucomatous TM cells using PANTHER. Key protein components from EV preparations were validated with Western blotting.

**Results:**

Results showed changes in the percentage of ECM proteins associated with EVs from glaucomatous TM cells compared to non-glaucomatous TM cells (5.7% vs 13.1% respectively). Correspondingly, we found that two ECM-related cargo proteins found across all samples, fibronectin and EDIL3 were significantly less abundant in glaucomatous EVs (<0.3 fold change across all groups) compared to non-glaucomatous EVs.

**Discussion:**

Overall, these data establish that ECM materials are prominent proteomic cargo in EVs from TM cells, and their binding to EVs is diminished in glaucoma.

## Introduction

Glaucoma, the second-most-common cause of blindness worldwide, is a neurodegenerative disease that culminates in the irreversible loss of retinal ganglion cells (RGCs) (1). Elevated levels of intraocular pressure (IOP) is one of the major risk factors for glaucoma disease progression and associated vision loss (2). As such, lowering IOP is currently the only effective treatment for glaucoma, utilizing both pharmacological and surgical methods. IOP is a function of aqueous humor (AH) production and its drainage through both conventional and unconventional (uveoscleral) outflow pathways. The conventional pathway is the source of resistance to unimpeded AH outflow, which determines IOP and is regulated by the cells that inhabit this pathway: the trabecular meshwork (TM), Schlemm’s canal (SC), and distal venous vessels (3–5).

The major source of resistance in the TM is the region known as the juxtacanalicular tissue (JCT), which is adjacent to the inner wall of SC (6, 7). The JCT is made up of ECM materials interspersed with TM cells, which have long cellular processes that communicate with both the inner wall (IW) endothelial cells and trabecular meshwork cells in the corneoscleral meshwork region (8–11). The ECM in this region is hydrated, allowing AH to move through the JCT and into the SC lumen. The ECM here is incredibly dynamic and composed of many different molecules that can influence outflow resistance, thereby regulating IOP. In fact, the continual remodeling of the ECM is comparable to a healing wound, and is thought to be part of an adaptive mechanism for IOP fluctuations (12). In support of this idea, the ECM components change in response to changes in IOP that impact the preferential flow pathways for AH (4, 13). Thus, flow through the TM is not uniform, but consists of low- and high-flow regions in which the TM expresses different ECM-related genes (14–16). In the glaucomatous TM, ECM dynamics and homeostasis is compromised, causing an excess of ECM to build up in the JCT region, creating increased resistance to outflow (16–18). The trigger for this ECM dysregulation is currently unknown and greater understanding of this is important to determine glaucomatous pathophysiology.

As in cancer cells that robustly regulate and maintain ECM, one likely mechanism for ECM regulation in the TM is via extracellular vesicles (EVs) [as reviewed in previous publications (19, 20)]. The EVs are nanoparticles that are released by every cell type and have a multitude of functions, one of which is ECM regulation (21, 22). As such, the EVs are released from TM cells *in vitro*, and from explanted TM tissue and are abundant in aqueous humor (21, 23–26). Moreover, the EVs play a role in ECM regulation by delivering both ECM protein cross-linkers and ECM proteases, such as matrix metalloproteinases (MMPs) and tissue inhibitors of metalloproteinases (TIMPs), and also by binding partially digested ECM materials (20, 27). We have previously shown that small extracellular vesicles (sEVs) released from TM cells in organ cultured explants bind fibronectin, and this process was disrupted following treatment with glucocorticoids (21). Significantly, patients exposed to high levels of glucocorticoids to treat retinal disease have a high incidence of ocular hypertension due to increased ECM materials in the TM (28, 29). Based on these studies, we hypothesized that the ECM binding profile and/or capacity of sEVs released from glaucomatous TM cells is altered compared with sEVs released from TM cells isolated from healthy eye donors. In this study, we compare the proteomic cargo of sEVs released from glaucomatous and non-glaucomatous TM cells *in vitro.*

## Methods

### Human trabecular meshwork cell culture

De-identified whole globes or corneal rims from human donors were obtained from Miracles in Sight (Winston-Salem, NC, USA) in accordance with the Declaration of Helsinki on research involving human tissue and with the approval of the Duke University Health System Institutional Review Board. The demographic characteristics of the human donors that contributed to this study are provided in **Table 1**. Human TM cells were isolated using a blunt dissection technique, characterized, and cultured in our laboratory as previously described (30, 31). For this study, nine separate TM strains were used: six isolated from non-glaucomatous human tissue and three isolated from glaucomatous human tissue between passages 3 and 6. For sEV collection, TM monolayers were differentiated in DMEM supplemented with 1% fetal bovine serum (FBS; Thermo Fisher Scientific, Waltham, MA, USA) and maintained by culturing in DMEM supplemented with 1% exosome-depleted FBS (Thermo Fisher Scientific) for approximately 90 days. The conditioned media from TM monolayers were collected every 48 h during media exchanges and stored at −80°C before further processing.

**Table 1:** Summary of donor information.

Cell strain	Age (years)	Sex	Race	Glaucoma
**TM120**	11 month	Male	Unknown	No
**TM134**	51	Male	White	No
**TM135**	77	Female	White	No
**TM144**	75	Female	White	No
**TM140**	60	Male	Black	No
**TM155**	58	Female	White	No
**TM201**	81	Female	White	Yes
**TM209**	71	Male	White	Yes
**TM211**	75	Female	White	Yes

### Small extracellular vesicle isolation

For this study, sEVs were isolated using a gentle double iodixanol (OptiPrep™; Sigma, USA) cushion ultracentrifugation, followed by an iodixanol cushioned-density gradient ultracentrifugation (C-DGUC), as described by Li et al. (32). In brief, collected conditioned media were centrifuged at 2,000 *g* for 10 minutes to remove cellular debris. The supernatants were centrifuged at 10,000 *g* for 30 minutes at 8°C. The resulting supernatant was carefully collected and layered onto a cushion of 60% iodixanol medium. Sedimented EVs were extracted from the iodixanol cushion interfaces and diluted using particle-free Dulbecco’s phosphate-buffered saline (PBS), layered over a 60% iodixanol cushion, and centrifuged at 100,000 *g*. The iodixanol cushion containing isolated sEVs was collected and used as the base layer for the iodixanol gradient. Density gradient ultracentrifugation was performed, and the medium was collected from the top in 1-mL increments to create 12 fractions. The fractions were then diluted in PBS and washed. The supernatant was discarded, and the remaining pellet containing purified EVs was resuspended in lysis buffer (100 mM tris, pH 6.8, 2% sodium dodecyl sulfate (SDS) and stored at −80°C until further analysis. The sEVs are typically found in fractions in a density range of approximately 1.07 g/mL–1.11 g/mL (33); however, we also detected the enrichment of EV markers in denser fractions and opted to assess these as a separate dataset.

### Sample preparation and LC-MS/MS analysis

For each sample, approximately 8 μg of total protein was used to prepare peptide mixtures for proteomic profiling. The proteins were cleaved with the trypsin/endoproteinase Lys-C mixture (V5072; Promega, Madison, WI, USA) using the paramagnetic beads-based method (34). Each digest was dissolved in 15 μL of 1%/2%/97% (by volume) of trifluoroacetic acid/acetonitrile/water solution, and 5 μL was injected into a 5 μm×5 mm PepMap™ Neo C18 column (Thermo Scientific™) in 1% acetonitrile in water for 3 minutes at a rate of 5 μL/minute. The analytical separation was then performed using an EasySpray PepMap Neo 75 μm × 150 mm, 2 μm, C18 column (Thermo Scientific) over 90 minutes at a flow rate of 0.3 μL/minute at 35°C using the Vanquish™ Neo ultra-high-performance liquid chromatography (UHPLC) system (Thermo Scientific). The 5%–35% mobile phase B gradient was used, where phase A was 0.1% formic acid in water and phase B was 0.1% formic acid in 80% acetonitrile. The peptides separated by LC were introduced into the Q Exactive™ HF Orbitrap mass spectrometer (Thermo Scientific) using positive electrospray ionization at 1900 V and with a capillary temperature of 275°C. The data collection was performed in the data-dependent acquisition (DDA) mode with 120,000 resolutions (at m/z 200) for MS1 precursor measurements. The MS1 analysis utilized a scan from 375 m/z to 1500 m/z, with a target automatic gain control (AGC) value of 1.0e6 ions, the radiofrequency (RF) lens set at 30%, and a maximum injection time of 50 ms. Advanced peak detection and internal calibration (EIC) were enabled during data acquisition. The peptides were selected for MS/MS using charge state filtering, monoisotopic peak detection, and a dynamic exclusion time of 25 seconds with a mass tolerance of 10 ppm. MS/MS was performed using higher-energy C-trap dissociation (HCD) with a collision energy of 30% ± 0.5%, detection in the ion trap using a rapid scanning rate, an AGC target value of 5.0e4 ions, a maximum injection time of 150 ms, and ion injection for all available parallelizable time enabled.

### Protein identification and quantification

For label-free relative protein quantification, raw mass spectral data files (.raw) were imported into Progenesis QI for Proteomics 4.2 software (Nonlinear Dynamics) for alignment of technical replicate data and peak area calculations. The peptides were identified using Mascot version 2.5.1 (Matrix Science) for searching the UniProt 2019-reviewed human database, which contains 20,243 entries. The Mascot search parameters were as follows: 10 ppm mass tolerance for precursor ions; 0.025 Da for fragment-ion mass tolerance; one missed cleavage by trypsin; a fixed modification of carbamidomethylation of cysteine; and a variable modification of oxidized methionine. Only the proteins identified with two or more peptides (i.e., Mascot scores > 15 for a peptide and > 50 for a protein corresponding to a protein confidence of *p* < 0.05), were included in the protein quantification analysis. To account for variations in experimental conditions and amounts of protein material in individual LC-MS/MS runs, the integrated peak area for each identified peptide was corrected using the factors calculated by the automatic Progenesis algorithm, utilizing the total intensities for all peaks in each run. The values representing protein amounts were calculated based on the sum of ion intensities for all identified constituent non-conflicting peptides. Protein abundances were averaged across the two duplicate runs for each sample.

### PANTHER analysis of the most abundant proteins

To assess the different protein classes, we used the Protein Analysis Through Evolutionary Relationships (PANTHER) software. We pooled the proteomic datasets from each biological replicate and sorted these by abundance. We then filtered out duplicate proteins to obtain the top 100 proteins from the pooled datasets. These were then inputted into the PANTHER website and analyzed for protein class. The data exported from this was then transferred to GraphPad Prism (GraphPad Software Inc., San Diego, CA, USA) to create charts used in figures.

**Figure 1:**
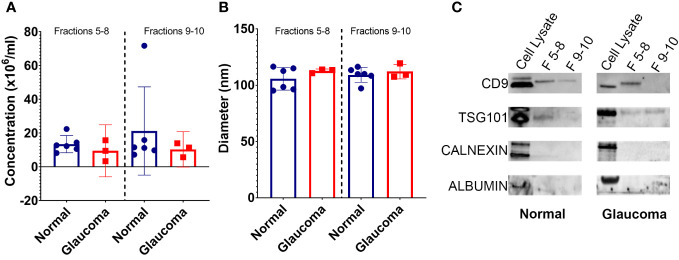
Properties of EVs isolated from glaucomatous and non-glaucomatous TM cells. **(A)** Concentration of EVs isolated from TM cells (10^6^ particles/mL) as measured by nanoparticle tracking analysis **(B)** Diameter of EVs isolated from TM cells as measured by nanoparticle tracking analysis. **(C)** Western blots showing EV markers CD9 and TSG101 in fractions 5-8 and 9-10, and negative EV control markers Calnexin and Albumin in cell lysate samples only.

### Western blotting

A standard Western blotting protocol was followed. Briefly, EV samples were solubilized in Laemmli buffer and approximately 5 µg of each sample was loaded onto SDS-polyacrylamide (PAGE) gels, separated electophoretically, and transferred to nitrocellulose membranes. The membranes were blocked with 5% bovine serum albumin (BSA) in tris-buffered saline with 0.01% Tween-20 (TBST) for 1 hour at room temperature on a rocking platform. After blocking, membranes were incubated with primary antibodies in blocking buffer at 4°C overnight. The antibodies used were CD9 (ab263019; Abcam, Cambridge, UK), TSG101 (ab125011; Abcam), Calnexin (ab133615; Abcam), albumin (ab207327; Abcam), epidermal growth factor (EGF)-like repeats and discoidin domains 3 (EDIL3; ab190692; Abcam), and fibronectin (ab6328; Abcam). Primary antibodies were removed, and membranes were washed three times for 10 minutes at room temperature in TBST. Horseradish peroxidase (HRP)-conjugated secondary antibodies [goat anti-mouse (#115-035-146) and goat anti-rabbit (#111-035-144); Jackson ImmunoResearch, West Grove, PA, USA] were added and incubated at room temperature for 1 hour. The membranes were washed again, as before, and developed using chemiluminescent reagents (SuperSignal™ West Atto; A38555; Thermo Fisher Scientific). The membranes were then imaged using the ChemiDoc™ Imaging System (BioRad) or the iBright Imaging System (Invitrogen). Protein band intensity was normalized to the concentration of particles (NTA) per protein (ug), which was measured by bicinchoninic acid (BCA) assay, as previously described (21).

### Nanoparticle tracking analysis

The ZetaView nanoparticle tracking analysis instrument (Particle Metrix, Ammersee, Germany) was used to determine exosome vesicle diameter and estimated particle concentration. For analysis, the instrument was calibrated for size using 100-nm polystyrene beads and the sample material was diluted to a concentration of 1: 5,000 in EV-free PBS. Averages of measurements taken in triplicate from eight positions within the imaging chamber at 25°C under a 405-nm laser were used to estimate vesicle size and concentration.

### Statistical analysis

Data are presented as the average (confidence interval range). The Student’s *t*-test was used to assess statistical significance between groups, with a *p*-value < 0.05 determined as being statistically significant.

## Results

### Characteristics of small extracellular vesicles released from TM cells

The EVs were isolated from conditioned media from glaucomatous TM (GTM) cells and non-glaucomatous or "normal" TM (NTM) cells. The gradient fractions with densities of approximately 1.05 g/mL–1.10 g/mL (fractions 5–8) and approximately 1.11 g/mL–1.17 g/mL (fractions 9–10) were analyzed.

Nanoparticle tracking analysis (NTA) was used to determine the size distribution and concentration of released EVs. The results showed no significant difference in the number of EVs released from the NTM and GTM samples—although there was some variability seen between the cell strains (**Figure 1A**). The size of EVs from both the NTM and GTM cells were within the expected size range (30 nm–150 nm) for sEVs and there was no significant difference in the size distribution between the groups (**Figure 1B**).

**Figure 2:**
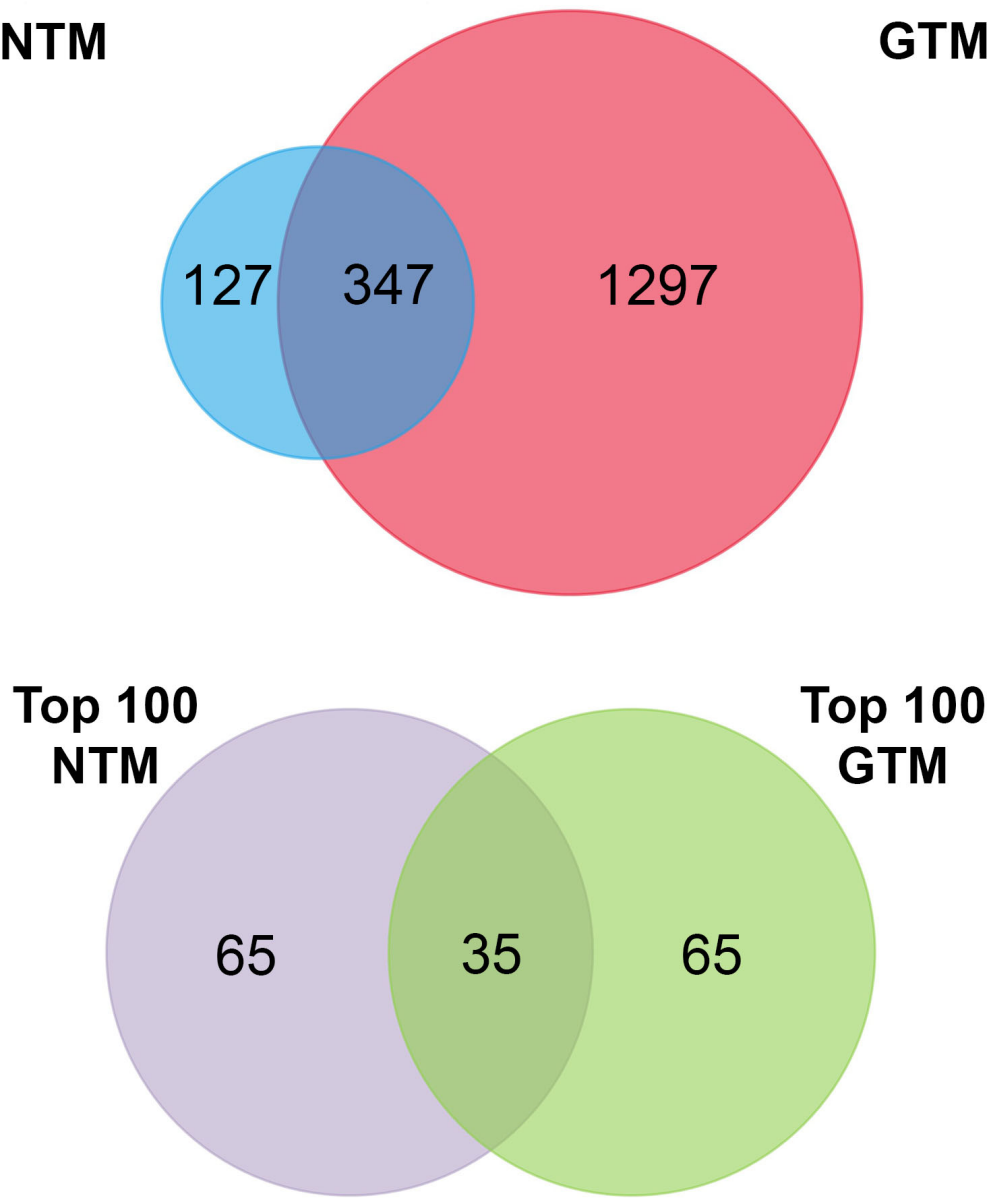
Venn diagrams summarizing the proteomic datasets. Top panel: all verified proteins from NTM and GTM EVs. Bottom panel: top 100 most abundant proteins in each dataset for further analysis. NTM, Normal Trabecular Meshwork; GTM, Glaucomatous Trabecular Meshwork.

Western blotting was conducted to determine the presence of EV markers CD9 and TSG101 in the EV preparations—both CD9 and TSG101 were found in fractions 5–8, but not as consistently in fractions 9 and 10 (**Figure 1C**). The preparations were negative for albumin and calnexin, indicating that they were free from cellular debris and thus pure sEV preparations (**Figure 1C**).

### Proteomic analysis of sEVs released from TM cells

To assess the proteomic cargo of EVs isolated from TM cells, we used mass spectrometry and validated target proteins by Western blotting. Mass spectrometry was conducted on isolated EVs and the top 100 most abundant proteins from the proteomic datasets were analyzed (**Figure 2**). The summaries of the top 100 proteins from the NTM EVs and the GTM EVs are presented in **Tables 2**, **3**, respectively. The complete proteomic datasets for the NTM and GTM groups are shown in **Supplementary Tables 1, 2**, respectively. The Venn diagrams demonstrate the distinct proteomic cargos of the NTM and GTM EVs—only 35 the 100 most abundant proteins in each group were shared between the groups. In both groups, fibronectin was the most abundant protein found on the sEVs from the TM cells. Many of the other most abundant proteins in each group were ECM or cytoskeleton related; however, they differed between groups. On the NTM sEVs, collagen isoforms, laminin isoforms, emilin, and other ECM proteins constituted the most abundant proteins. In contrast, on the GTM sEVs, fibrillin, plectin, and actins were the most abundant proteins. Collectively, this demonstrates the presence of ECM glycoproteins on NTM sEVs, compared with cytoskeleton- and actin-related proteins on GTM sEVs.

**Figure 3:**
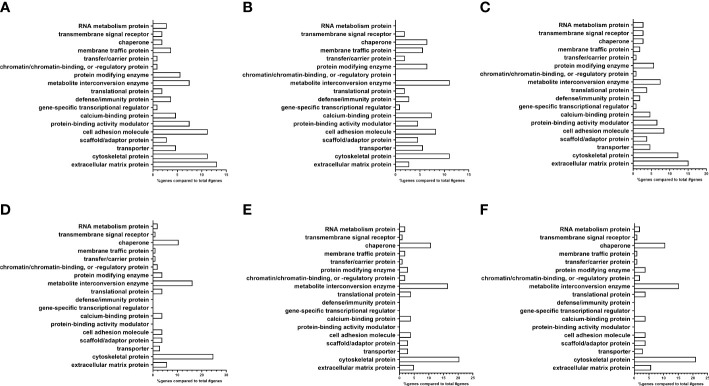
Analysis of proteomic dataset by protein class showing differences in ECM proteins between EV subpopulations. **(A–C)** Non-glaucomatous TM EVs. **(A)** Pooled fractions 5–10; **(B)** pooled fractions 5–8; and **(C)** pooled fractions 9 and 10. **(D–F)** Glaucomatous TM EVs. **(D)** Pooled fractions 5–10; **(E)** pooled fractions 5–8; and **(F)** pooled fractions 9 and 10.

**Table 2:** 100 most abundant proteins from NTM EVs.

Protein name	Gene symbol	Unique spectral count
Fibronectin	*FN1*	511
Basement membrane-specific heparan sulfate proteoglycan core protein	*HSPG2*	211
Collagen alpha-1(I) chain	*COL1A1*	89
Collagen alpha-1(III) chain	*COL3A1*	85
Alpha-2-macroglobulin	*A2M*	63
Collagen alpha-2(I) chain	*COL1A2*	57
Desmoplakin	*DSP*	52
Collagen alpha-3(VI) chain	*COL6A3*	47
Laminin subunit gamma-1	*LAMC1*	35
Laminin subunit alpha-5	*LAMA5*	33
Collagen alpha-1(XII)	*COL12A1*	33
Filamin-A	*FLNA*	32
Actin, cytoplasmic 1	*ACTB*	32
Agrin	*AGRN*	32
Annexin A2	*ANXA2*	29
Major vault protein	*MVP*	29
EMILIN-1	*EMILIN1*	29
Lactadherin	*MFGE8*	28
Myoferlin	*MYOF*	28
Galectin-3-binding protein	*LGALS3BP*	27
Lysyl oxidase homolog 2	*LOXL2*	27
Tenascin	*TNC*	26
EGF-like repeat and discoidin I-like domain-containing protein 3	*EDIL3*	26
Alpha-1-antiproteinase	*SERPINA1*	23
Integrin beta 1	*ITGB1*	23
Junction plakoglobin	*JUP*	23
Thrombospondin-1	*THBS1*	22
Laminin subunit beta-1	*LAMB1*	21
Moesin	*MSN*	21
Laminin subunit alpha-4	*LAMA4*	20
Laminin subunit beta-2	*LAMB2*	20
Annexin A6	*ANXA6*	19
Heat shock cognate 71-kDa protein	*HSPA8*	19
Brain acid-soluble protein 1	*BASP1*	18
Prostaglandin F2 receptor negative regulator	*PTGFRN*	18
Syntenin 1	*SDCBP*	17
Pyruvate kinase PKM	*PKM*	16
Sodium-/potassium-transporting ATPase subunit alpha-1	*ATP1A1*	16
Serum albumin	*ALB*	16
Glyceraldehyde-3-phosphate dehydrogenase	*GAPDH*	15
Nidogen-2	*NID2*	15
Prelamin-A/C	*LMNA*	15
Prolyl endopeptidase FAP	*FAP*	14
Periostin	*POSTN*	14
Stomatin-like protein 3	*STOM*	14
14-3-3 protein sigma	*SFN*	14
5′-nucleotidase	*NT5E*	14
Fructose-bisphosphate aldolase A	*ALDOA*	13
Alpha-enolase	*ENO1*	13
Programmed cell death 6-interacting protein	*PDCD6IP*	13
Voltage-dependent calcium channel subunit alpha 2/delta-1	*CACNA2D1*	13
Tubulointerstitial nephritis antigen like	*TINAGL1*	13
Collagen alpha-1(VI) chain	*COL6A1*	13
Intercellular adhesion molecule 1	*ICAM1*	13
Dipeptidyl peptidase 4	*DPP4*	12
Annexin A1	*ANXA1*	12
Plasma membrane calcium-transporting ATPase 4	*ATP2B4*	12
Actin, aortic smooth muscle	*ACTA2*	12
Annexin A5	*ANXA5*	12
Nidogen-1	*NID1*	12
Heat shock protein (HSP) 90-beta	*HSP90AB1*	12
Elongation factor 1-alpha 1	*EEF1A1*	12
Tubulin beta-2B chain	*TUBB2B*	12
Integrin alpha-3	*ITGA3*	11
Integrin alpha-7	*ITGA7*	11
Integrin alpha-V	*ITGAV*	11
Cell surface glycoprotein MUC18	*MCAM*	11
Histone H4	*HIST1H4A*	11
Histone H2B type 1-K	*HIST1H2BK*	11
Eukaryotic initiation factor 4A-I	*EIF4A1*	11
ATP synthase subunit beta, mitochondrial	*ATP5F1B*	11
HLA class I histocompatibility antigen, B alpha chain	*HLA-B*	10
Vimentin	*VIM*	10
Integrin alpha-2	*ITGA2*	10
EH domain-containing protein 1	*EHD1*	10
Ras-related protein Rab-7a	*RAB7A*	10
Neuroblast differentiation-associated protein AHNAK	*AHNAK*	10
Alpha-2-HS-glycoprotein	*AHSG*	10
Pentraxin-related protein PTX3	*PTX3*	10
Myosin-9	*MYH9*	10
Glia-derived nexin	*SERPINE2*	10
Polyadenylate-binding protein 1	*PABPC1*	10
CD44	*CD44*	9
Target of Nesh-SH3	*ABI3BP*	9
Myristoylated alanine-rich C-kinase substrate	*MARCKS*	9
Collagen alpha-1(XVIII)	*COL18A1*	9
Collagen alpha-2(VI) chain	*COL6A2*	9
Programmed cell death protein 6	*MME*	9
Tubulin alpha-1B chain	*TUBA1B*	9
Elongation factor 2	*EEF2*	9
Synaptic vesicle membrane protein VAT-1 homolog	*VAT1*	8
Guanine nucleotide-binding protein G(I)/G(S)/G(T) subunit beta-1	*GNB1*	8
Guanine nucleotide-binding protein G(i) subunit alpha-2	*GNAI2*	8
Ras-related protein R-Ras	*RRAS*	8
Unconventional myosin-Ic	*MYO1C*	8
EH domain-containing protein 2	*EHD2*	8
Basigin	*BSG*	8
Immunoglobulin superfamily member 8	*IGSF8*	8
Vinculin	*VCL*	8
Hornerin	*HRNR*	8

**Table 3:** 100 most abundant proteins from GTM EVs.

Protein name	Gene symbol	Unique spectral count
Fibronectin	*FN1*	295
Plectin	*PLEC*	293
Neuroblast differentiation-associated protein AHNAK	*AHNAK*	229
Filamin-A	*FLNA*	168
Basement membrane-specific heparan sulfate proteoglycan core protein	*HSPG2*	158
Fibrillin-1	*FBN1*	148
Myosin-9	*MYH9*	147
Vimentin	*VIM*	128
Talin-1	*TLN1*	100
Prelamin-A/C	*LMNA*	100
Myoferlin	*MYOF*	80
Actin—cytoplasmic 1	*ACTB*	78
Annexin A2	*ANXA2*	78
Myosin-10	*MYH10*	76
Caldesmon	*CALD1*	73
Alpha-actinin-1	*ACTN1*	66
Prolow-density lipoprotein receptor-related protein 1	*LRP1*	66
Annexin A6	*ANXA6*	65
Pyruvate kinase PKM	*PKM*	64
Keratin, type II cytoskeletal 1	*KRT1*	58
Actin, alpha cardiac muscle 1	*ACTC1*	58
Cytoplasmic dynein 1 heavy chain 1	*DYNC1H1*	56
Endoplasmin	*HSP90B1*	52
Annexin A5	*ANXA5*	51
Elongation factor 2	*EEF2*	50
Glyceraldehyde-3-phosphate dehydrogenase	*GAPDH*	50
Filamin-C	*FLNC*	49
Clathrin heavy chain 1	*CLTC*	49
Spectrin alpha chain, non-erythrocytic 1	*SPTAN1*	46
Elongation factor 1-alpha 1	*EEF1A1*	45
Alpha-enolase	*ENO1*	44
Transitional endoplasmic reticulum ATPase	*VCP*	44
Vinculin	*VCL*	44
Alpha-actinin-4	*ACTN4*	44
Collagen alpha-1(XII) chain	*COL12A1*	43
Tubulin alpha-1B chain	*TUBA1B*	42
Annexin A1	*ANXA1*	42
Cytoskeleton-associated protein 4	*CKAP4*	41
Unconventional myosin-Ic	*MYO1C*	41
EMILIN-1	*EMILIN1*	40
Endoplasmic reticulum chaperone BiP	*HSPA5*	40
Ribosome-binding protein 1	*RRBP1*	39
Superoxide dismutase [Mn], mitochondrial	*SOD2*	38
Protein disulfide-isomerase A3	*PDIA3*	38
ATP synthase subunit beta, mitochondrial	*ATP5F1B*	37
Collagen alpha-3(VI) chain	*COL6A3*	36
Hemicentin 1	*HMCN1*	36
Kinectin	*KTN1*	36
Keratin, type I cytoskeletal 9	*KRT9*	35
Heat shock cognate 71-kDa protein	*HSPA8*	34
Protein disulfide isomerase	*P4HB*	34
Trifunctional enzyme subunit alpha, mitochondrial	*HADHA*	34
Transketolase	*TKT*	33
Fructose-bisphosphate aldolase A	*ALDOA*	33
ATP synthase subunit alpha, mitochondrial	*ATP5F1A*	33
Spectrin beta chain, non-erythrocytic 1	*SPTBN1*	33
60S ribosomal protein L4	*RPL4*	33
NADH-cytochrome b5 reductase 3	*CYB5R3*	32
Dysferlin	*DYSF*	32
Dolichyl-diphosphooligosaccharide—protein glycosyltransferase subunit 1	*RPN1*	31
Protein AHNAK2	*AHNAK2*	31
Heterogeneous nuclear ribonucleoproteins A2/B1	*HNRNPA2B1*	30
Myosin-11	*MYH11*	30
Keratin, type I cytoskeletal 10	*KRT10*	29
Keratin, type II cytoskeletal 2 epidermal	*KRT2*	29
Filamin-B	*FLNB*	29
Collagen alpha-1(I) chain	*COL1A1*	29
Major vault protein	*MVP*	29
Histone H4	*H4C1*	29
Stress-70 protein, mitochondrial	*HSPA9*	29
MICOS complex subunit MIC60	*IMMT*	28
Neutral alpha-glucosidase AB	*GANAB*	28
Aldehyde dehydrogenase X, mitochondrial	*ALDH1B1*	28
Vigilin	*HDLBP*	27
Staphylococcal nuclease domain-containing protein 1	*SND1*	27
Histone H2B type F-S	*H2BFS*	27
Band 4.1-like protein 2	*EPB41L2*	27
Catenin alpha-1	*CTNNA1*	27
Heat shock protein (HSP) 90-alpha	*HSP90AA1*	26
UDP-glucose 6-dehydrogenase	*UGDH*	26
Ubiquitin-like modifier-activating enzyme 1	*UBA1*	26
Heat shock protein (HSP) 90 beta	*HSP90AB1*	26
Heat shock 70-kDa protein 1A	*HSPA1A*	26
Procollagen-lysine,2-oxoglutarate 5-dioxygenase 2	*PLOD2*	26
Integrin beta 1	*ITGB1*	26
Calnexin	*CANX*	26
5′-nucleotidase	*NT5E*	26
Heterogeneous nuclear ribonucleoprotein M	*HNRNPM*	25
Fatty acid synthase	*FASN*	25
EGF-like repeat and discoidin I-like domain-containing protein 3	*EDIL3*	25
Calreticulin	*CALR*	25
L-lactate dehydrogenase A chain	*LDHA*	25
Integrin alpha-V	*ITGAV*	25
Glutaminase kidney isoform, mitochondrial	*GLS*	25
60S ribosomal protein L6	*RPL6*	25
LIM domain-only protein 7	*LMO7*	25
Adenylyl cyclase-associated protein 1	*CAP1*	24
Prolyl 4-hydroxylase subunit alpha-2	*P4HA2*	24
Trifunctional enzyme subunit beta, mitochondrial	*HADHB*	24
Collagen alpha-2(I) chain	*COL1A2*	24

### PANTHER analysis of 100 most abundant proteins in normal and glaucoma TM sEVs

Next, using the PANTHER database, we determined the protein class differences between the NTM and GTM groups (**Figure 3**) (35, 36). We analyzed the differences between all isolated sEVs (**Figures 3A, D**), as well as separating each of our sEV sub-populations into fractions 5–8 (**Figures 3B, E**) and fractions 9 and10 (**Figures 3C, F**).

From the PANTHER database, the protein class analysis in the total NTM vs. GTM dataset (**Figures 3A, D**) showed a lower percentage of extracellular matrix proteins associated with sEVs from GTM cells than from NTM cells (5.7% vs. 13.1%, respectively). In the sEV subpopulation consisting of fractions 5–8 (**Figures 3B, E**), there was an increased percentage of ECM proteins found in the GTM EVs compared with the NTM EVs (4.9% vs. 2.8%). In the sEV subpopulation consisting of fractions 9 and 10 (**Figures 3C, F**), there was a decreased percentage of ECM proteins associated with the GTM EVs compared with the NTM EVs (5.7% vs. 15.1%).

There was also a lower percentage of cell adhesion molecules (CAMs) on the GTM EVs than on the NTM EVs (3.8% vs. 11.2%) This was also demonstrated in the 5–8 sEV fraction group (GTM 3.9% vs. NTM 8.3%) and in the 9 and 10 fraction group (GTM 3.8% vs. NTM 8.5%).

However, the GTM EVs had a higher percentage of cytoskeletal proteins associated with them than NTM EVs (24.7% vs. 11.2%, respectively). This increase in cytoskeletal proteins was maintained in both the 5–8 sEV subpopulation (GTM 20.4% vs. NTM 11.1%) and in the 9 and 10 sEV subpopulation (GTM 21.0% vs. NTM 12.3%).

### Decreased levels of fibronectin and EDIL3 associated with sEV from GTM cells

Two of the most abundant proteins found in sEVs across all biological replicates from NTM and GTM cells were fibronectin and EDIL3. Fibronectin is a major component of the ECM in the TM (37), and EDIL3 is a ligand of integrin αV/β3 that promotes endothelial cell adhesion and migration, promotes epithelial-to-mesenchymal transition, and is associated with both endothelial cells and the extracellular matrix (38–44). When validating these proteins, we used two unique populations of EVs from both the NTM and GTM groups—sEVs from fractions 5–8 and sEVs from fractions 9 and 10.

**Figure 4:**
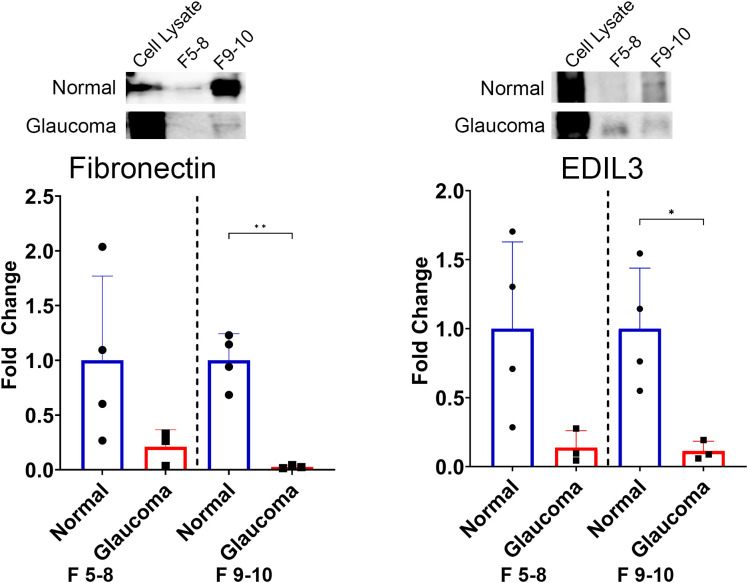
Differences in the expression of candidate ECM proteins between non-glaucomatous and glaucomatous EVs. Fibronectin is decreased in glaucomatous EVs compared with non-glaucomatous EVs. EDIL3 is decreased in glaucomatous EVs compared with non-glaucomatous EVs. The band intensity was normalized to the number of particles in each sample. *p < 0.05, **p < 0.01, Student’s *t*-test. F, fractions.

We validated the presence of these ECM-related proteins within the EV cargo by Western blotting. Quantification of the bands was conducted, and the band intensity was normalized to the number of particles per sample. **Figure 4** shows a decrease in Fn associated with EVs from GTM compared with EVs from NTM cells in both EV populations, i.e., fractions 5–8 [fold change 0.21 (−0.18, 0.6); *p* = 0.1476] and fractions 9 and 10 [0.03 (−0.01, 0.06); *p* = 0.0011]. **Figure 4** shows the decreased abundance of EDIL3 in EVs from GTM cells compared with NTM cells in both EV populations, i.e., fractions 5–8 [0.14 (−0.16, 0.44); *p* = 0.0707] and fractions 9–10 [0.11 (−0.06, 0.29); *p* = 0.0195].

## Discussion

The current study rigorously profiled the proteome of sEVs from GTM and NTM cells. Although we observed some similarities, the overall proteomic profiles of the sEV cargo from the NTM and GTM cells were very different. Specifically, there were decreased numbers of ECM proteins in the GTM sEVs. The two major ECM proteins, fibronectin (37) and EDIL3 (38), were significantly decreased in the GTM sEVs compared with the NTM sEVs. Taken together, our *in vitro* findings were consistent with the aberrant accumulation of ECM materials in glaucoma *in vivo*, which likely contributes to increased outflow resistance, and, therefore, increased IOP.

Although we investigated sEV released from TM cells specifically, it is highly possible that the sEVs released from other cells in the eye may also affect the TM tissue as the AH moves through the conventional outflow pathway and exits the eye. One such potential source of sEVs is the non-pigmented ciliary epithelium, which has been shown to release sEVs and influence ECM remodeling in the TM (45). This element is a limitation of the current study as we did not examine the effects of non-TM sEVs on TM cells.

When assessing the proteomics dataset, we only examined the 100 most abundant proteins present in each of our sEV populations, as lower-abundance proteins were more likely to represent contaminating proteins. For this analysis, the PANTHER database was utilized to assess the classes of proteins associated with the sEV populations—we separately analyzed the total sEV population and the two separate subpopulations. This was in order to sufficiently compare the sEVs that we were able to isolate from the TM cells, as different sEV populations released from the cells may perform different functions in physiologically normal and diseased states. In line with previous studies, we found that there was a decreased number of ECM proteins associated with sEVs from GTM cells when compared with those associated with NTM sEVs (21). This was true for both the overall sEV population and the fractions 9 and 10 sub-population—both showed approximately 50% less ECM proteins in the GTM sEV group. There were also more CAMs found on the sEVs from NTM cells. CAMs are involved with ECM protein binding, which is a likely mechanism for the differences in ECM regulation/protein binding by sEVs. The results indicate that the different sEV populations have different functions within the TM.

Based on previous studies, we examined the binding of fibronectin with the GTM sEVs—fibronectin has previously been shown to be a highly abundant protein on TM sEVs (21, 23). In the presence of dexamethasone, a steroid that induces a glaucoma phenotype *in vitro* and is known to cause elevated IOP and glaucoma in patients, there were decreased levels of fibronectin in the TM sEVs (21). Consistent with these data, our study showed that fibronectin was one of the most abundant proteins across our TM sEV samples; however, it was decreased in the GTM sEVs compared with the NTM sEVs. Although fibronectin showed a decrease in both subpopulations of sEVs, the decrease only reached significance in the sEVs from the subpopulation comprising fractions 9 and 10. This may be because of the small number of biological replicates used—we used only three POAG cell strains and compared this with four non-glaucoma cell strains by Western blotting. Our hypothesis is that the binding capacity of sEVs from GTM cells is altered, and, therefore, these sEVs did not bind fibronectin to deliver proteases such as MMPs (46) to degrade it, or to target it for phagocytosis by TM cells. If sEVs from GTM cells are not contributing to fibronectin degradation, excess fibronectin will be found in the TM contributing to the ECM buildup and increased outflow resistance. Significantly, to our knowledge, there are no other studies that have compared sEVs from NTM and GTM cells. Instead, previous studies examined sEVs from NTM cells using single cell strains with technical replicates, or up to six biologically independent cell strains (23, 47, 48).

An abundant protein observed in all samples was EDIL3—an ECM protein involved in endothelial cell adhesion, migration, and angiogenesis when bound with integrins (38–40). EDIL3 is also associated with epithelial–mesenchymal transition, which is indicative of a more fibrotic phenotype and environment, and is linked to the transforming growth factor beta (TGFβ) signaling pathway (41–43). TGFβ is a key driver of ECM production and fibrosis in the TM and TGFβ2 has been shown to be increased in the aqueous humor of patients with glaucoma (49–52). The results here show a decrease in EDIL3 in GTM sEVs compared with NTM sEVs, indicating that there may be more EDIL3 present in the GTM owing to it not being removed via sEVs. Zhang et al. showed that depletion of EDIL3 suppressed the proliferation and migration of lens epithelial cells, decreased the expression of α-smooth muscle actin and vimentin, and decreased Smad2 and Smad3 phosphorylation (43). In glaucoma, α-smooth muscle actin and vimentin are altered and can be induced by TGFβ (53, 54). Increased levels of EDIL3 in the TM cells would likely cause increased expression of profibrotic factors, such as α-smooth muscle actin and vimentin, and elements of the TGFβ pathway, which may lead to increased ECM accumulation in the TM cells owing to TGFβ activity.

Finally, when looking at the proteomic datasets overall, it is apparent that the protein cargo of sEVs differs greatly between the GTM and NTM groups. Out of the top 100 most abundant proteins, there were only 35 overlapping proteins between the two groups, and across the whole proteome, only approximately 350 proteins overlapped. Of these, many were ribosomal proteins and extracellular matrix proteins. This shows that sEVs play different roles in diseased and non-diseased cells. Furthermore, the most abundant ECM proteins are quite different between the two groups, which also demonstrates that their roles in ECM homeostasis are also distinct from each other.

Taken together, the data presented here indicate that the sEVs released from TM cells likely play a role in ECM binding and thus turnover in the conventional outflow pathway. The significant differences in the ECM profiles of sEVs from TM cells isolated from healthy versus glaucomatous donor eyes suggest that dysfunctional binding and opsonization of the ECM in glaucomatous eyes may contribute to decreased ECM degradation in the TM, ultimately leading to increased IOP.

## Data availability statement

The mass spectrometry proteomics data have been deposited to the ProteomeXchange Consortium via the PRIDE (55) partner repository with the dataset identifier PXD044913 and 10.6019/PXD044913.

## Ethics statement

De-identified human donor whole globe or corneal rims were obtained from Miracles in Sight (Winston-Salem, NC) in accordance with the Declaration of Helsinki for research involving human tissue and with the Duke University Health System Institutional Review Board approval.

## Author contributions

FM: Conceptualization, Data curation, Formal analysis, Funding acquisition, Investigation, Methodology, Project administration, Supervision, Writing – original draft, Writing – review & editing. BR: Data curation, Formal analysis, Investigation, Writing – review & editing. HR: Data curation, Investigation, Writing – review & editing. NS: Data curation, Formal analysis, Investigation, Methodology, Writing – review & editing. WS: Conceptualization, Funding acquisition, Project administration, Resources, Supervision, Writing – review & editing.

## Supplementary material

Supplementary Figure 1 Sample of ZetaView output report showing particle size (*x*-axis) and concentration (*y*-axis).

Supplementary Figure 2 All analyzed Western blots for fibronectin and EDIL3. N1, N2, N3, and N4 are samples from independent normal donors. G1, G2, and G3 are samples from independent glaucoma donors.

Supplementary Table All NTM and GTM EV proteins.
