# Supplementary figures and images for: Comparison of the extracellular vesicle proteome between glaucoma and non-glaucoma trabecular meshwork cells
Source: Front Ophthalmol (Lausanne). Author manuscript; Available in PMC 2024 Jan 12. (PMC10785745; doi:10.3389/fopht.2023.1257737)

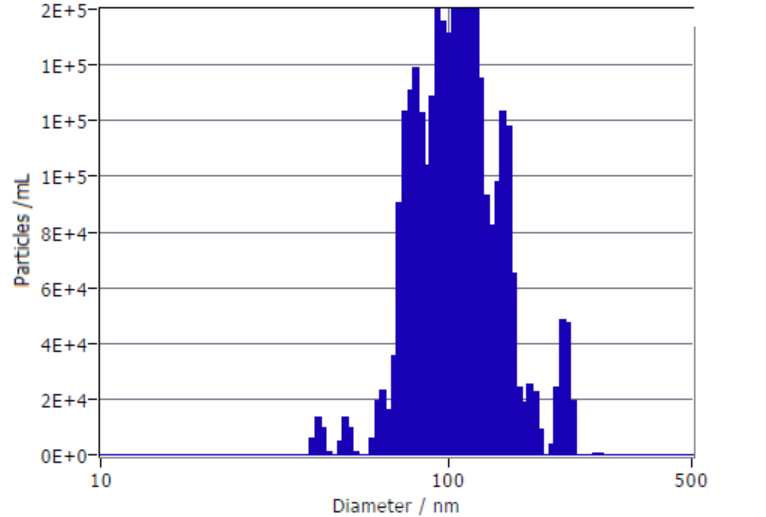

Supplement: Supp Fig 1 — SUPPLEMENTARY FIGURE 1 Sample of ZetaView output report showing particle size (x-axis) and concentration (y-axis). [file NIHMS1956271-supplement-Supp_Fig_1.tif]

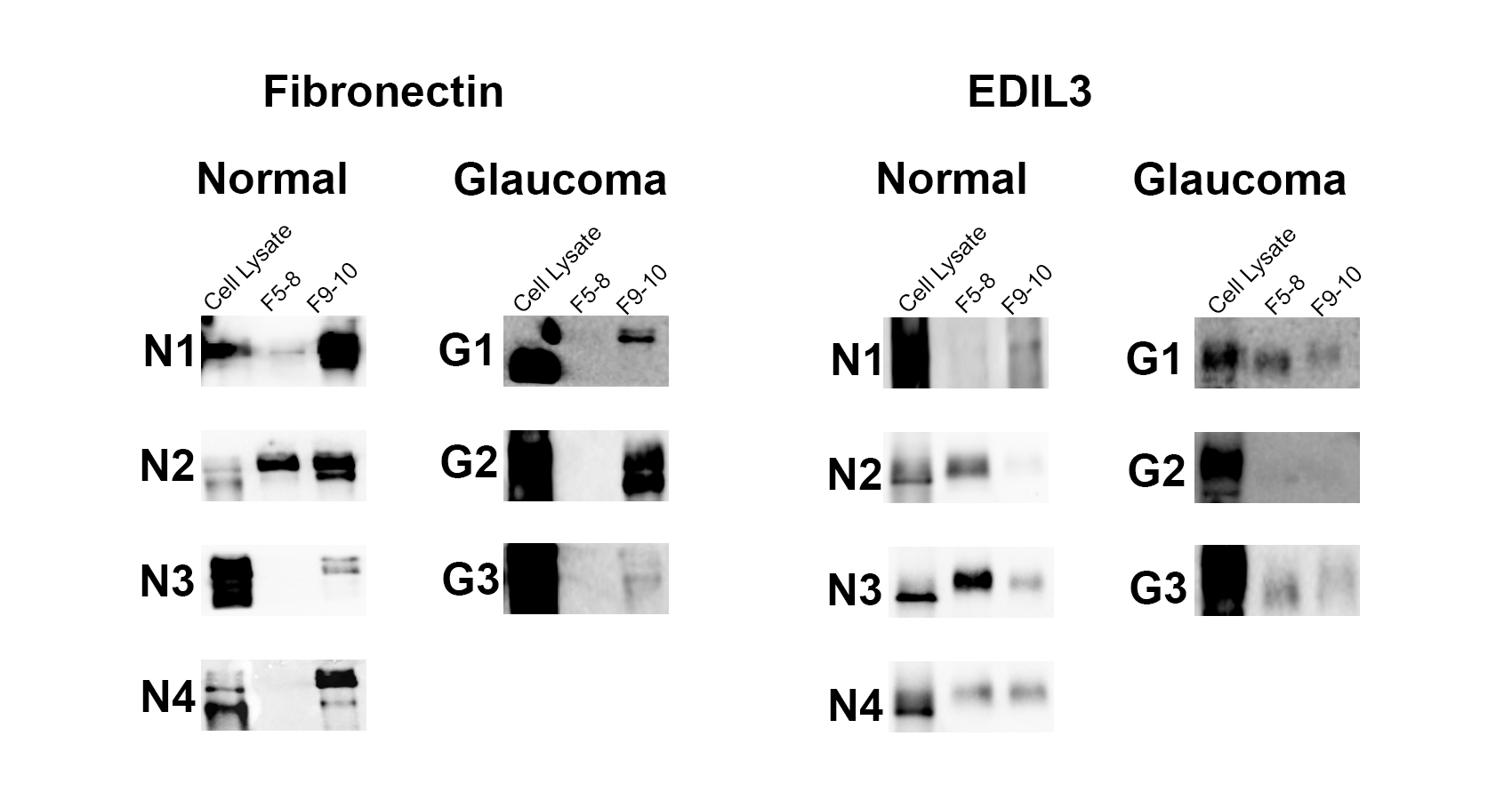

Supplement: Supp Fig 2 — SUPPLEMENTARY FIGURE 2 All analyzed Western blots for fibronectin and EDIL3. N1, N2, N3, and N4 are samples from independent normal donors. G1, G2, and G3 are samples from independent glaucoma donors. [file NIHMS1956271-supplement-Supp_Fig_2.tif]
